# The Molecular Basis and Therapeutic Aspects of Cisplatin Resistance in Oral Squamous Cell Carcinoma

**DOI:** 10.3389/fonc.2021.761379

**Published:** 2021-10-22

**Authors:** Yali Cheng, Shaoming Li, Ling Gao, Keqian Zhi, Wenhao Ren

**Affiliations:** ^1^ Department of Oral and Maxillofacial Surgery, The Affiliated Hospital of Qingdao University, Qingdao, China; ^2^ School of Stomatology of Qingdao University, Qingdao, China; ^3^ Key Lab of Oral Clinical Medicine, The Affiliated Hospital of Qingdao University, Qingdao, China

**Keywords:** oral squamous cell carcinoma, cisplatin, chemoresistance, molecular mechanisms, cancer therapy

## Abstract

Oral squamous cell carcinoma (OSCC) is a kind of malignant tumors with low survival rate and prone to have early metastasis and recurrence. Cisplatin is an alkylating agent which induces DNA damage through the formation of cisplatin-DNA adducts, leading to cell cycle arrest and apoptosis. In the management of advanced OSCC, cisplatin-based chemotherapy or chemoradiotherapy has been considered as the first-line treatment. Unfortunately, only a portion of OSCC patients can benefit from cisplatin treatment, both inherent resistance and acquired resistance greatly limit the efficacy of cisplatin and even cause treatment failure. Herein, this review outline the underlying mechanisms of cisplatin resistance in OSCC from the aspects of DNA damage and repair, epigenetic regulation, transport processes, programmed cell death and tumor microenvironment. In addition, this review summarizes the strategies applicable to overcome cisplatin resistance, which can provide new ideas to improve the clinical therapeutic outcome of OSCC.

## 1 Introduction

Oral cancer is the sixth leading cause of global cancer-related deaths (1), with the most common type being oral squamous cell carcinoma (OSCC). OSCC usually presents in smokers and drinkers aged 40 to 70 years, and in recent years human papillomavirus (HPV) infection has also been identified as a major cause (2, 3). OSCC tends to have early, extensive lymph node metastases and is among the malignancies with low survival rates. Despite advances in diagnostic and therapeutic approaches for OSCC in the past decades, its five-year survival rate remains suboptimal (4). At present, early stage non-metastatic OSCC (stages I and II) can be largely cured by surgery alone, but for advanced OSCC (stages III and IV), besides the standard surgical treatment and external radiotherapy, supportive treatment with a combination of chemotherapeutic agents is required (5).

**Figure 1:**
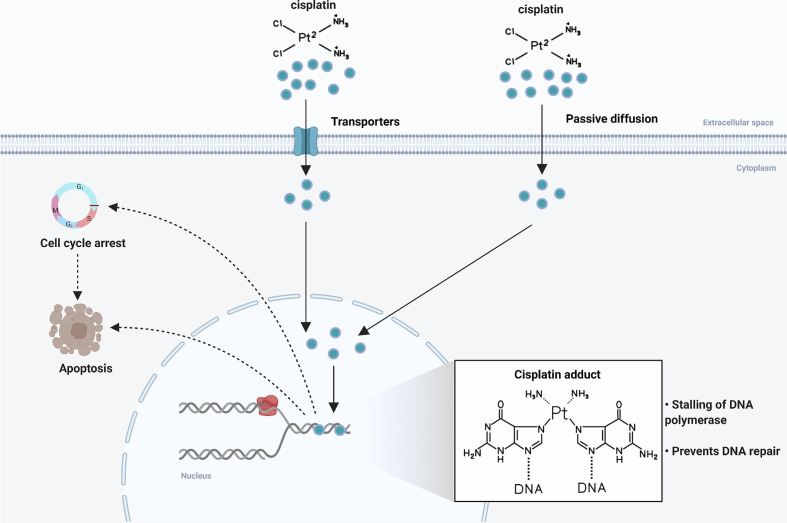
Mechanism of action of cisplatin. Following entry into tumor cells by active transport or passive diffusion, cisplatin forms DNA adducts in the nucleus, leading to cell cycle arrest and cell apoptosis.

Cisplatin (cis-diaminedichloroplatinum, CDDP), a chemotherapeutic agent with high antitumor activity against many cancers in clinical application, is the first-line and most widely used chemotherapeutic drug for OSCC. In 1968, cisplatin was first discovered to have the ability to lead to tumor regression in a tumor-bearing mouse model (6), and its antitumor activity was confirmed in a variety of solid tumors over the next decade (7–9). The cytotoxic effect of cisplatin is mainly manifested as forming adducts with genomic DNA, which directly damages DNA and inhibits DNA replication, thereby arresting the cell cycle and eventually leading to cell death (**Figure 1**) (10). In a large-scale randomized clinical trial, a treatment regimen of postoperative radiotherapy combined with cisplatin chemotherapy significantly reduced local and regional recurrences and prolonged disease-free survival (DFS) in patients with advanced OSCC (11). Meanwhile, preoperative intraarterial induction chemotherapy with cisplatin partially reduced tumor volume and improved overall prognosis (12). Considering the side effects and chemoresistance of cisplatin, researchers have developed thousands of cisplatin analogues (13), but only carboplatin and oxaliplatin are currently approved for clinical use. And these platinum-based agents, such as carboplatin, are less effective than cisplatin at the same dose, although they reduce side effects to some extent (14). In addition, other anti-cancer drugs included targeted drugs also do not have the absolute advantages. A phase III clinical trial conducted in Sweden reported that cetuximab plus radiotherapy (RT) produced an overall toxicity comparable to cisplatin plus RT in patients with locoregionally advanced head and neck squamous cell carcinoma (HNSCC), but showed inferiority in terms of local tumor control and treatment prognosis (15). Therefore, cisplatin remains the cornerstone of OSCC chemotherapy.

In recent years, with the widespread use of platinum-based chemotherapeutic agents, especially cisplatin, many patients have experienced serious toxic side effects. In particular, the development of chemoresistance has severely attenuated the efficacy of cisplatin in the treatment of OSCC. To our knowledge, no review has systematically summarized the mechanisms of cisplatin resistance in OSCC. This paper provides an overview regarding the mechanisms of cisplatin resistance in OSCC based on now-available research findings and summarizes promising strategies to overcome cisplatin resistance in OSCC.

## 2 Molecular Basis of Cisplatin Resistance in OSCC

### 2.1 DNA Damage Response and Cisplatin Resistance in OSCC

The combination of DNA damage tolerance and DNA damage repair is critical for tumor cells to counteract cisplatin-induced DNA damage. The major forms of DNA damage repair are mis-match repair (MMR), interstrand cross-link repair (ICR), nucleotide excision repair (NER), base-excision repair (BER), homologous recombination repair (HR), trans-damaged DNA synthesis (TLS) and non-homologous end joining (NHEJ) (16). Enhanced NER has been reported to be associated with cisplatin-resistant phenotypes in a variety of cancers (17). ERCC1 is an important member of the NER pathway, and its expression has been demonstrated to be negatively correlate with the efficacy of platinum-based chemotherapy and the prognosis of several cancers (18–20), including OSCC. For example, ERCC1 expression was enhanced in advanced HNSCC patients who responded poorly to cisplatin-based chemoradiotherapy and had the habit of chewing areca nuts (21); Snail-mediated upregulation of ERCC1 led to cisplatin resistance in OSCC (22). Furthermore, not only the expression level of ERCC1 can regulate the response of tumor cells to cisplatin, but ERCC1 gene polymorphisms have also been proved to make sense. Avinash Tejasvi et al. reported that ERCC1 C118T genotype were more frequently detected in OSCC specimens and that patients carrying this genotype had a worse response to cisplatin (23).

TLS has also been found involved in cisplatin resistance in OSCC. Polη is known to be a DNA polymerase that functions in the TLS pathway, which can bypass the cisplatin-DNA adducts formed during cisplatin chemotherapy and maintain DNA synthesis, thus resisting cisplatin-induced DNA damage. Therefore, endogenous Polη levels may be a potent marker of the efficacy of cisplatin (24). Chen et al. reported that in OSCC, prolonged endoplasmic reticulum (ER) stress upregulated Polη expression and induced the development of cisplatin resistance, whereas the presence or absence of ER stress had little effect on the response to cisplatin of cells lacking POLH, the gene encoding Polη. Interestingly, this study also found that p53 nuclear translocation occurred in endoplasmic reticulum stress-adapted cells and that intracellular cisplatin uptake was not significantly different from control cells, but the accumulation of cisplatin-induced DNA damage was dramatically reduced. Once p53 was knocked down, the cells could hardly tolerate prolonged ER stress and cisplatin treatment (25). This suggested that prolonged ER stress might counteract the effects of cisplatin by repairing DNA damage through the Polη-dependent TLS pathway, and inducing p53 nuclear translocation to make cells tolerate DNA damage. In summary, targeting DNA repair pathways may be a promising approach to enhance chemotherapy sensitivity. However, these findings have not been validated in clinical practice.

### 2.2 Epigenetic Mechanisms and Cisplatin Resistance in OSCC

The epigenetic modifications include several different forms such as methylation, histone modification, and regulation of non-coding RNAs. Genetic mechanisms and epigenetic mechanisms influence each other and work together to obtain the characteristics of cancer (26). Many pieces of evidence indicate that epigenetic mechanisms are also involved in the occurrence and development of OSCC, including cisplatin resistance in OSCC (**Table 1**).

**Table 1:** Epigenetic mechanisms and cisplatin resistance in OSCC.

Molecules	Expression	Targets and signaling pathways	Regulatory outcomes	Reference
Non-coding RNAs				
circRNAs				
circ_0109291	Up	miR-188-3p/ABCB1	Promoted proliferation and inhibited apoptosis	(27)
circ_0001971	Up	miR-194/miR-204	Promoted proliferation, migration, invasion and inhibited apoptosis	(28)
lncRNAs				
OIP5-AS1	Up	miR-27b-3p/TRIM14	Inhibited apoptosis	(29)
MALAT1	Up	PI3K/AKT/m-TOR	Inhibited apoptosis	(30)
LHFPL3-AS1	Up	miR-362-5p/CHSY1	Promoted proliferation, migration, invasion and inhibited apoptosis	(31)
ZFAS1	Up	miR-421/MEIS2	Inhibited apoptosis	(32)
CASC2	Down	miR-31-5p/KANK1	Enhanced chemoresistance	(33)
PVT1	Up	miR-194-5p/HIF1a	Enhanced proliferation and cisplatin resistance	(34)
XIST	Up	miR-27b-3p	Promoted proliferation, inhibited apoptosis and enhanced CDDP resistance	(35)
CYTOR	Up	miR-1252-5p/miR-3148/LPP	Induced EMT and resistance to cisplatin	(36)
HOXA11-AS	Up	miR-214-3p/PIM1	Promoted proliferation and inhibited apoptosis	(37)
HOTAIR	Up	–	Enhanced proliferation and cisplatin resistance	(38)
ANRIL	Up	MRP1 and ABCC2	Promoted proliferation, inhibited apoptosis and suppressed cisplatin cytotoxicity	(39)
UCA1	Up	miR-184/SF1	Promoted proliferation, inhibited apoptosis and enhanced cisplatin resistance	(40)
miRNAs				
miR-5787	Down	MT-CO3	Promoted cisplatin resistance, affected oxidative phosphorylation and aerobic glycolysis.	(41)
miR-132	Down	TGF-β1	Promoted migration and invasion and decreased chemosensitivity	(42)
miR-21	Up	–	Enhanced oncogenicity and chemoresistance of OSCC cells	(43)
miR-654-5p	Up	GRAP/Ras/Erk	Promoted proliferation, metastasis, and chemoresistance	(44)
miR-1246	Up	CCNG2	Promoted cancer stemness and drug resistance	(45)
miR-485-5p	Down	PAK1	Induced EMT and cisplatin resistance	(46)
miR-203	Down	PIK3CA/Akt	Inhibited apoptosis	(47)
miR-372	Up	ZBTB7A/TRAIL-R2	Enhanced oncogenic potential and cisplatin resistance	(48)
miR-27b	Down	FZD7/beta-catenin	Promoted proliferation, migration and suppressed cisplatin sensitivity	(49)
miR-125b	Down	PRXL2A/NRF2	Enhanced drug resistance	(50)
miR-222	Up	PUMA	Drived the oncogenesis and enhanced chemoresistance	(51)
Methylation				
DDX3	Up	FOXM1/NANOG	Inhibited cell death and enhanced CSC-like features	(52)
Bax	Down	p53/Akt	Inhibited cell death	(53)
LCN2	Up	NE-kappa B	Inhibited apoptosis and induced cisplatin resistance	(54)

#### 2.2.1 Non-Coding RNA-Based Mechanisms

Non-coding RNAs participate in extensive physiological and pathological processes. Increasing evidences indicate that non-coding RNAs, including circular non-coding RNAs (circRNAs), long non-coding RNAs (lncRNAs) and microRNAs (miRNAs), play a vital role in cisplatin resistance of OSCC.

Circ_0109291 had higher expressions in CDDP-resistant OSCC tissues and cells compared with CDDP-sensitive OSCC tissues and cells, and mechanistically promoted proliferation but inhibited the apoptosis of OSCC cells *via* miR-188-3p/ABCB1 axis (27). LncRNAs act in a similar form to circRNAs. For instance, long non-coding RNA Opa-interacting protein 5 antisense RNA 1 (OIP5-AS1) sponged miR-27b-3p and then influenced the expression of TRIM14, and knockdown of OIP5-AS1 restored the CDDP sensitivity of resistant OSCC cells (29). Futhermore, lncRNAs can modulate chemoresistance of OSCC *via* influencing epithelial mesenchymal transition (EMT). LncRNA MALAT1 functionally reduced apoptotic cell death by promoting EMT process and activating the PI3K/AKT/m-TOR signaling pathway in cisplatin-resistant OSCC cells (30). Studies of differentially expressed circRNAs or lncRNAs associated with OSCC cisplatin resistance have been emerging. However, these findings seemed to be cell-specific and could not be validated in other OSCC cell lines or clinical samples.

MicroRNAs (miRNAs) can negatively regulate gene expression. Yu et al. reported the difference of miRNAs profile between the cisplatin-resistant TSCC cell lines and their parental cell lines for the first time. Compared with parental cells, there were 19 differential miRNAs in resistant cells, among which 17 were upregulated and 2 were downregulated. In further studies, researchers found that silence of miR-214 and miR-23a or overexpression of miR-21 could reverse chemoresistance against cisplatin in cisplatin-resistant subline (55). MiR-5787 was found downregulated in cisplatin-resistant TSCC cells and functionally target mitochondrial cytochrome c oxidase subunit 3 (MT-CO3). Upregulation of miR-5787 in cisplatin-resistant cells and knockdown its expression in parental cells could regulate the responses to cisplatin of two cell lines respectively, and these results were verified in further *in vivo* experiments (41).

**Figure 2:**
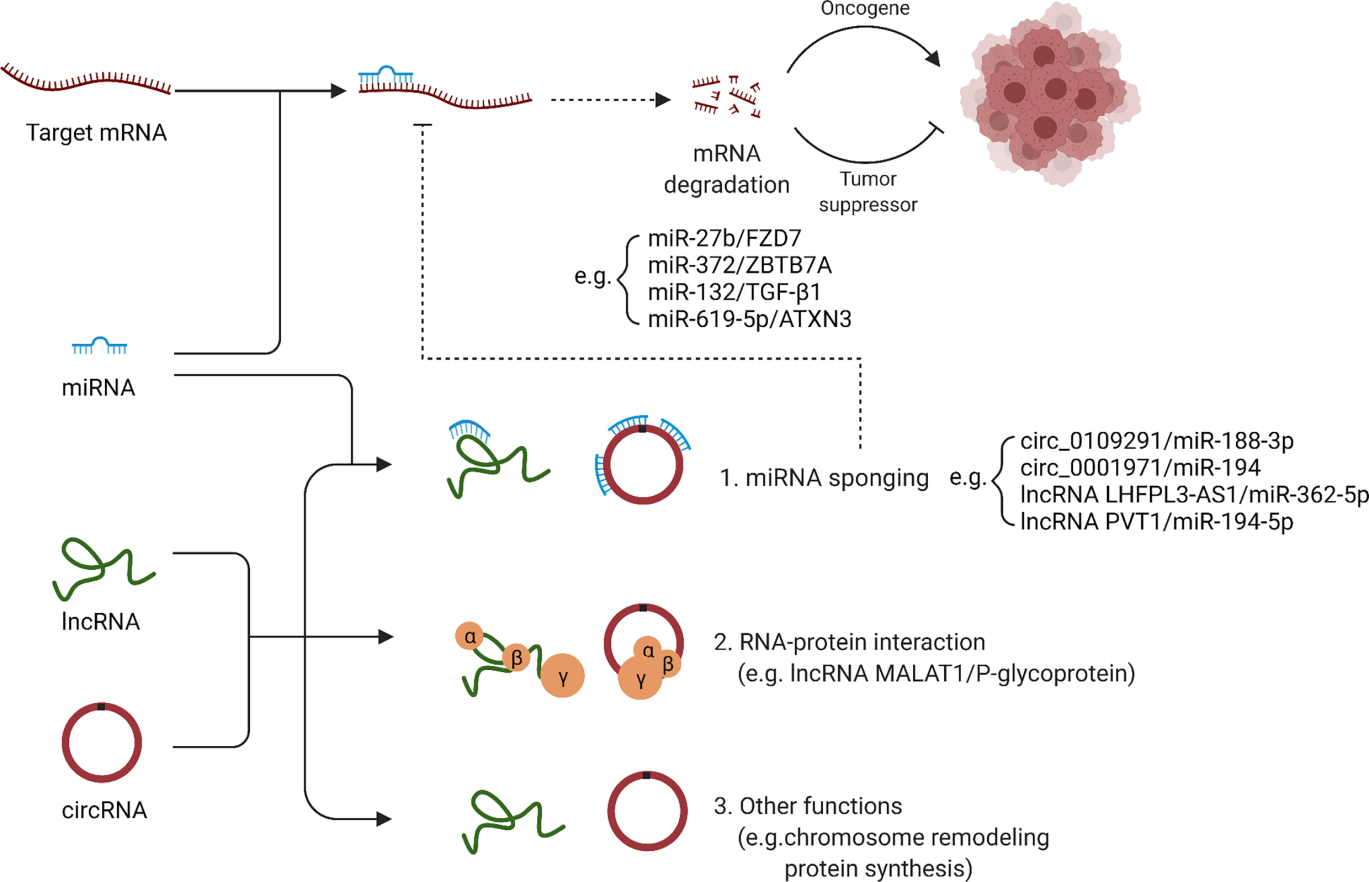
Regulation of cisplatin resistance by ncRNAs: a molecular mechanism. LncRNAs and circRNAs can function as sponges for miRNAs, and thus affect the regulatory role of miRNAs on target genes. In addition, lncRNAs and circRNAs can also interact with proteins, remodel chromosome structure and affect protein synthesis.

Previous studies on the non-coding RNAs involved in OSCC cisplatin resistance have mainly focused on function of circRNAs and lncRNAs as sponges for miRNAs and the regulatory role of miRNAs on target genes (**Figure 2**). Since non-coding RNAs have other complex functions that have been less studied so far, their association with cisplatin resistance of OSCC should be further revealed in the future.

#### 2.2.2 Methylation

Methylation is one of the components of epigenetic regulation and the most common methylation modifications are DNA methylation, RNA methylation and histone methylation. Methylation can cause genome instability and mutation, and then make the cancer cells acquire malignant characteristics (56). Wang et al. found the expression level of ten-eleven translocation 1 (TET1) was associated with cisplatin resistance, stem cell properties and o6-methylguanine-DNA methyltransferase (MGMT) methylation in OSCC. The use of TET1-siRNA induced MGMT promoter methylation and cell apoptosis, thereby enhancing the cisplatin sensitivity of OSCC cells with stemness (57). DDX3, a human DEAD-box RNA helicase associated with lymph node metastasis, cell migration and invasion, was found upregulated in cisplatin-resistant OSCC cells and chemotherapy-non-responder OSCC patients. Knockdown of DDX3 restored cisplatin-induced cell death in chemotherapy-resistant cell lines and reduced the proportion of cells with cancer stem cells (CSCs)-like features *via* suppressing the expression of FOXM1 and NANOG. What’s more, DDX3 could regulate m^6^A demethylase ALKBH5 directly, which resulted in decreased m^6^A methylation in FOXM1 and NANOG nascent transcript that contribute to cisplatin resistance in OSCC (52). Therefore, DDX3 was expected to be an effective therapeutic target to overcome cisplatin resistance in OSCC. Together, these studies suggest that cisplatin resistance in OSCC may be associated with methylation of cancer-related genes, and further studies of methylation may be valuable for tracing chemoresistance.

### 2.3 Programmed Cell Death (PCD) and Cisplatin Resistance in OSCC

Cell death is an indispensable process for maintaining the normal state of living organisms, and can be divided into accidental cell death (ACD) and regulatory cell death (RCD), while RCD is also called programmed cell death (PCD). At present, more and more new forms of PCD other than apoptosis have been discovered, and they are found to be involved in various pathological processes. Emerging evidence suggests that PCD, especially apoptosis and autophagy, is involved in cisplatin resistance in OSCC.

#### 2.3.1 Autophagy

Over the past few decades, researchers have made remarkable breakthroughs with regard to comprehension on what role autophagy plays in systemic health and disease, in particular the recognition that autophagy could inhibit or promote tumor growth and respond to anti-cancer treatments (58–60). Therefore, studies about the role of autophagy will contribute to the development of innovative therapies.

It has been shown that DNA-damaging chemotherapeutic agents such as cisplatin, carboplatin, and 5-fluorouracil could induce autophagy, thereby reducing apoptosis and making tumor cells resistant to chemotherapy. Li et al. found high autophagic flux in cisplatin-resistant OSCC cells, which showed as increased conversion rate of autophagic protein LC3, decreased expression of p62, and increased autophagosomes that were visible by transmission electron microscope (61). High positive rates of CD44, ABCB1, ADAM17 were observed in clinical OSCC specimens and cisplatin-resistant OSCC cells. Interestingly, enhanced autophagy and mitophagy were also detected. After the use of autophagy inhibitors, decreased expression of CD44, ABCB1, ADAM17 and increased sensitivity of cisplatin-resistant OSCC cells to cisplatin could be detected (62). Reactive Oxygen Species (ROS) and cancer-associated fibroblasts (CAFs) have been reported to be associated with tumor progression in OSCC, and studies showed that these two factors also contributed to cisplatin resistance through the induction of autophagy (63, 64).

These results have demonstrated that enhanced autophagy led to cisplatin resistance of OSCC, and inhibition of autophagy might be an effective method to reverse chemotherapy resistance. However, autophagy plays as a double-edged sword in the development of cancer (65, 66). In terms of drug resistance, a study showed that inhibition of autophagy led to cisplatin resistance in laryngeal squamous cell carcinoma (LSCC) (67). Whether autophagy is negatively associated with sisplatin resistance in OSCC remains unknown and requires further investigation.

#### 2.3.2 Apoptosis

Apoptosis is a form of PCD regulated by intrinsic and extrinsic pathways and activated when cells are attacked by special factors like DNA damage (68). During tumorigenesis and progression, cancer cells have to carry anti-apoptotic proteins to maintain cell viability. Nowadays, chemotherapeutic agents mostly rely on triggering apoptosis to induce cell death. Alternations in apoptosis related molecules and pathways would make anti-cancer drugs could not function at conventional concentrations, which means drug resistance.

The Bcl-2 family consists of anti-apoptotic proteins (e.g., Bcl-2 and Bcl-xL) and pro-apoptotic proteins (e.g., Bax, Bak, and Bad). It has been demonstrated that the expressions of Bcl-2-family members have a non-negligible effect on the response of cancers to chemotherapy (69). For example, Bcl-xL was overexpressed in cisplatin-resistant OSCC cell lines and suppression on its upstream regulators to attenuate Bcl-xL expression could promote apoptosis (70). Mcl-1 is also an important member of the Bcl-2 anti-apoptotic family. A survey about the expression of Bcl-2 anti-apoptotic proteins in 68 human cancer cell lines showed that in many solid tumors, the expression level of Mcl-1 was much higher than that of other Bcl-2 anti-apoptotic members (71). Maji et al. found that the Mcl-1 expression was upregulated in both chemoresistant OSCC lines and chemoresistant tumors. Mechanistically, STAT3 and AKT-mediated GSK3β both functioned in regulating the expression of Mcl-1. After blocking Mcl-1 expression by applying siRNA or chemical inhibitors, a significant increase in apoptosis could be observed, suggesting that overexpressed Mcl-1 was related to multiple drug resistance in OSCC (72). Beyond that, reduced expression of pro-apoptotic proteins also contributed to chemoresistance. Bax expression was reduced in cancer tissues of OSCC patients who were resistant to cisplatin-based chemoradiatherapy and two cisplatin-resistant OSCC cells lines. Bax was a downstream gene in p53/Akt pathway, the application of Akt inhibitors released the inhibition of Bax and increased the proportion of apoptosis among cisplatin-resistant OSCC cells (53). In summary, restoration of the inhibited apoptotic process may be an effective strategy to induce OSCC cell death.

Survivin is a member of the inhibitor of apoptosis protein (IAP) family, and recognized as a potential predictive biomarker for cancers for its high expression (73, 74). Münscher et al. found survivin was expressed at high levels in HNSCC, and positively correlated with the malignant characteristics (75). Another study reported that nicotine reduced cisplatin-induced apoptosis in oral cancer cells, while this protective effect could be attenuated when survivin was inhibited (76). YM155 is a small molecule inhibitor of survivin that has been found to be effective and selective in recent years. Experiments showed that, YM155 induced apoptosis and autophagic cell death in OSCC cells by effectively downregulating survivin in the cytoplasm and upregulating Beclin1 through the Akt/mTOR pathway (77). Meanwhile, the combination of YM155 with cisplatin yielded better anti-cancer effects than monotherapy (78). More recently, an antagonist of IAPs, Debio 1143, has shown potential to enhance the antitumor activity of cisplatin and radiotherapy in HNSCC, and a phase III clinical trial will be conducted to demonstrate its efficacy (79).

In general, targeting anti-apoptotic molecules or promoting the expression of pro-apoptotic molecules may be an effective mechanism to reverse chemoresistance in OSCC.

### 2.4 The Tumor Microenvironment (TME) and Cisplatin Resistance in OSCC

**Figure 3:**
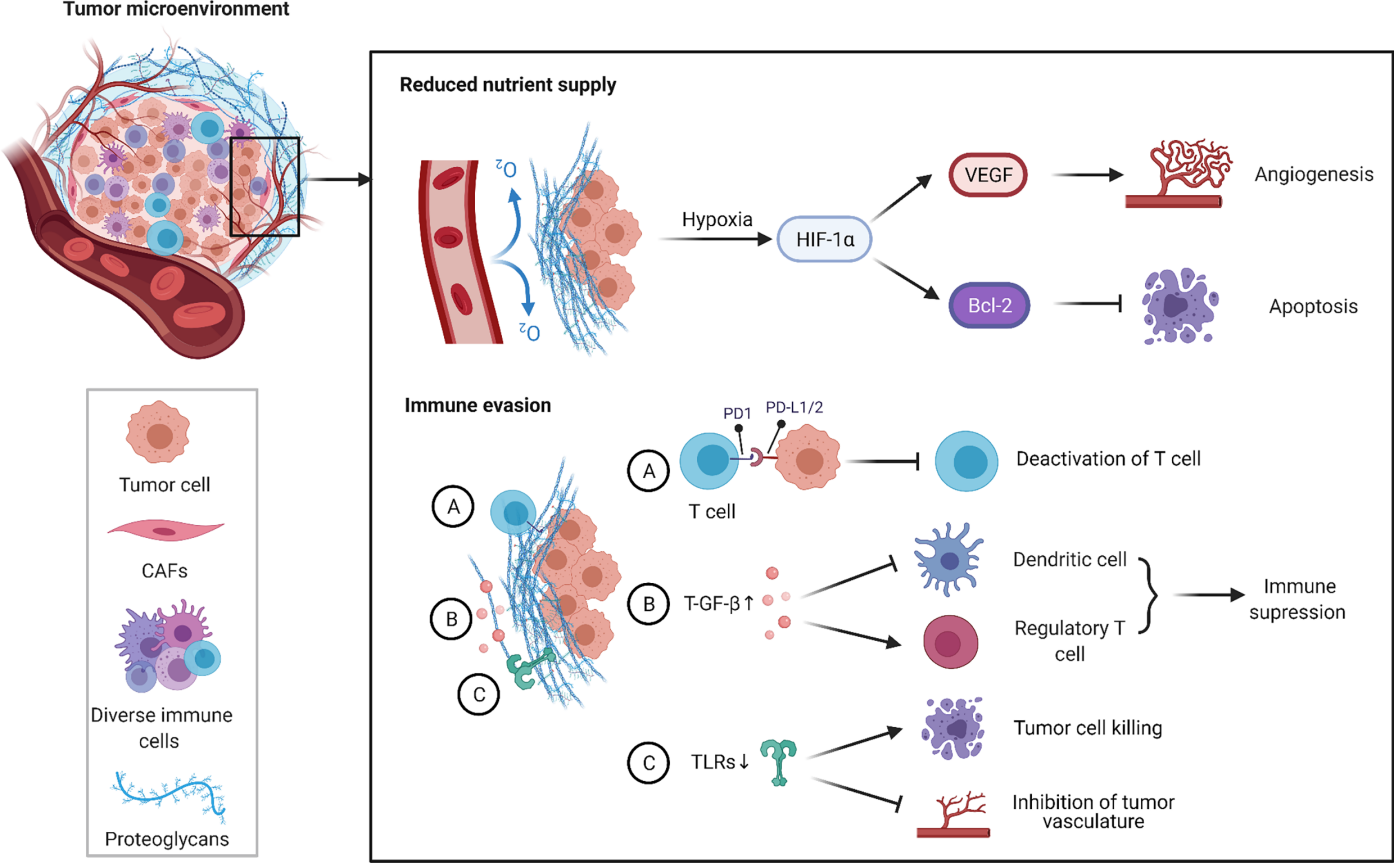
TME and cisplatin resistance in OSCC. TME is typically characterized by hypoxia and immunosuppression. Hypoxia induces HIF-1α-mediated signaling cascades, including induction of VEGF expression to promote tumor angiogenesis in hypoxic conditions and inhibition of apoptosis by regulating the expression of Bcl-2 anti-apoptotic family. In addition, a large number of cellular factors released by cancer cells and various bystander cells cause immune evasion by regulating the production and function of immune cells. Overall, the above pathways contribute to the cisplatin resistance of OSCC.

The tumor microenvironment consists of the tumor cells themselves, multiple stromal cells and extracellular matrix, and is usually characterized by hypoxia, and immunosuppression (80–82). The interactions of various cells and cytokines form a complex network of mechanisms (83), and recent studies have revealed that TME is responsible for the induction of multidrug resistance in OSCC (**Figure 3**).

#### 2.4.1 Hypoxic Microenvironment

OSCC is a solid tumor with a high hypoxia degree inside and this hypoxic condition may cause patients respond poorly to anticancer treatments (84). Hypoxia can activate the expression of HIFs to maintain cell viability, especially HIF-1α, which is widely expressed in different cancer cells (85). High expression of HIF-1α has been demonstrated to be associated with the malignant features of OSCC, and patients with high-expression levels of HIF-1α tend to have a shorter overall survival (86). HIF-1α can activate the transcription of multiple genes under hypoxic conditions, including vascular endothelial growth factor (VEGF) (87). To some extent, HIF-1α promotes angiogenesis in tumors under hypoxic environment by inducing the expression of VEGF, thereby regulating the balance of oxygen supply and metabolism within the tumor, and improving the survival of tumor cells (88). Moreover, HIF-1α participates in the regulation of apoptosis. For example, HIF-1α inhibited cell apoptosis by promoting the expression of the anti-apoptotic protein Bcl-2 while suppressing the expression of the pro-apoptotic proteins Bax and Bak, and the application of siRNA to downregulate HIF-1α expression restored sensitivity to chemoradiotherapy of OSCC cells (89, 90). Qi et al. reported that under hypoxic conditions, the synergistic application of metformin and cisplatin suppressed the expression of the upstream transcription factor NK-κB of HIF-1α, thereby blocking the synthesis of HIF-1α to enable cisplatin to exert its anticancer effects without difficulty (91). These studies validate the relationship between HIF-1α and resistance to cisplatin, suggesting that hypoxia significantly affects cisplatin therapy and further investigation of potential molecular mechanisms in hypoxia-induced chemoresistance will provide new promising directions for the treatment of OSCC.

#### 2.4.2 Immune Microenvironment

In recent years, most HNSCC have been shown to have highly immunosuppressive TME which allows the tumor to escape the host immune response. What’s more, tumor immune escape is also a crucial contributor for the formation of multi drug resistance, because drug-resistant cells are more likely to evade recognition and killing by the immune system than drug-sensitive cells (92). Programmed death 1 (PD-1) is widely expressed on the surface of immune cells such as T cells, B cells and macrophages, whereas programmed death ligand 1 (PD-L) is expressed in a variety of tumor cells. The combination of PD-L and PD-1 on the surface of T cells can inhibit the activity of killer T cells and even induce their apoptosis, thus allowing tumor cells to gain immune escape (93). A global proteomic profiling of cisplatin-sensitive and cisplatin-resistant OSCC cell lines showed CMTM6 was the top-ranked upregulated protein in cisplatin-resistant cells (94). What’s more, it had been demonstrated that in HNSCC, CMTM6 positively correlated with PD-L1 expression and both of CMTM6 and PD-L1 were associated with a worse prognosis. Knockdown of CMTM6 resulted in decreased PD-L1 expression and increased tumor-infiltrating CD4+CD8+ T cells (Tregs), which consequently improved antitumor immunity and cisplatin-induced apoptosis; whereas overexpression of CMTM6 resulted in increased PD-L1 expression and decreased sensitivity of OSCC cells to cisplatin (95, 96). The relevance of PD-L1, another ligand of PD-1, to cisplatin resistance in OSCC has also been reported. Sudo, et al. established a cisplatin-resistant OSCC cell line, HSC-2, and found that cisplatin upregulated the gene expression levels of PD-L2, ABCG2 in HSC-2 in a time-dependent manner. In addition, STAT1/3 mediated the induction of PD-L2 by cisplatin, and PD-L2-positive cells have higher metastatic and invasive potential compared to PD-L2-negative cells (97). These studies suggested that anti-PD-L immunotherapy may be valuable for cisplatin-resistant OSCC patients.

TGF-β is an important regulator of immune homeostasis in tumor microenvironment, and it can create an immunosuppressive TME by regulating the production and function of multiple immune cells (such as regulatory T cells and dendritic cells) (98). In OSCC, the application of miRNAs mimics targeting TGF-β1 effectively reduced the gene expression of TGF-β1, inhibited growth, invasion, and metastasis of tumor cells and increased the sensitivity of drug-resistant cells to cisplatin (42). Based on this study, we can speculate that the high expression of TGF-β1 may lead to cisplatin resistance in OSCC by suppressing immune cell function.

In the tumor microenvironment, aberrant expression of Toll-like receptors (TLRs) not only gives tumor cells the opportunity to escape the host immune response, but also contributes to tumor development by promoting proliferation and angiogenesis and resisting apoptosis (99, 100). Current preclinical trials have shown that monotherapy or combination therapeutic strategies of applying TLRs modulators can effectively improve tumor prognosis (101). In OSCC, the application of Poly(I:C), an agonist of TLR3, reduced drug efflux by inhibiting ABC transporters in tumor cells, activated immune cells in TME, induced delayed tumor cell apoptosis that based on caspase3 pathway, and enhanced the anti-cancer effect of low-dose cisplatin with reduced adverse side effects (102). This chemoimmunotherapeutic strategy of sequential application of the TLR3 agonist Poly(I:C) and low-dose cisplatin was suitable for long-term treatment of OSCC patients with poor physical status and bad response to conventional chemotherapy drugs.

Understanding the role played by immune checkpoints and the tumor microenvironment in anticancer therapy could help improve single-drug-based chemotherapy regimens and thus facilitate the development of a novel chemotherapy strategy that incorporates immunotherapy.

### 2.5 Transport and Cisplatin Resistance in OSCC

**Figure 4:**
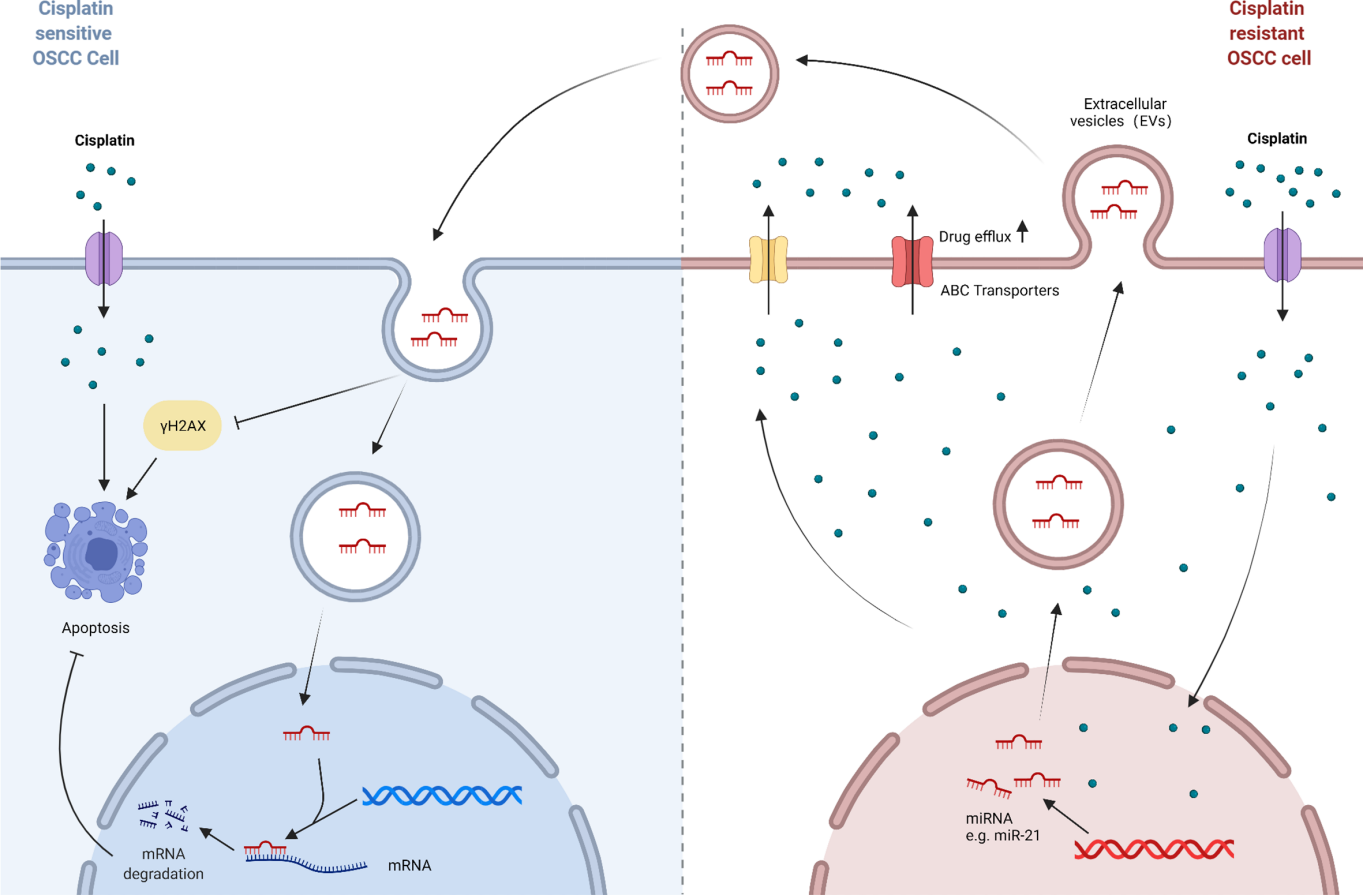
A model of transport process-mediated cisplatin resistance in OSCC. ABC transporters act as transmembrane proteins that result in the efflux of cisplatin from OSCC cells and reduce intracellular drug levels. And extracellular vesicles act as natural carriers of biological signals that can deliver substances such as functional RNAs from cisplatin-resistant OSCC cells to parental cells, thus enabling the propagation of cisplatin resistance in OSCC cells.

Anti-cancer drugs must reach an appropriate concentration at the target site in order to exert their killing effect in tumor cells. Predictably, if the concentration in cells does not reach the concentration required for treatment for some reason, the anti-cancer drugs will not function, in which case drug resistance will occur. ATP binding cassette (ABC) transporters are well known for their role to enable drug efflux in multidrug resistance (MDR). They serve as transmembrane proteins that facilitate the efflux of anticancer drugs from cancer cells hence reducing intracellular drug levels. In addition, extracellular vesicles also play a key role in cancer chemoresistance as natural carriers of signals in the organism. For example, exosomes can wrap therapeutic drugs and export them out of the cell, or transport drug efflux pumps into the cell (103). The mechanisms of cisplatin resistance based on transport process in OSCC are reviewed below (**Figure 4**).

#### 2.5.1 ABC Transporters

Currently, the most studied ABC transporters associated with drug resistance include ABCB1 (P-glycoprotein/P-gp/MDR1), ABCG2 (Breast Cancer Resistance Protein/BCRP) and ABCC1 (Multidrug Resistance Protein1/MRP1) (104). However, the regulatory roles of ABC transporters in cisplatin-resistant OSCC are still poorly understood.

Choi, et al. established three cisplatin-resistant OSCC cell lines and found elevated gene expression of BCRP and MDR1 in these three cell lines (105). Lu et al. found higher expression of ABCC1 and ABCG2 in specimens from OSCC patients who had poor differentiation and received chemotherapy compared to those from patients with well differentiation and was not treated with chemotherapy. In addition, *in vitro* experiments revealed that the Hedgehog signaling pathway was activated in OSCC cells resistant to cisplatin and 5-FU. Overexpression of SHH, a key gene in the Hh pathway, resulted in increased ABCG2 expression, which suggested that the Hh pathway promoted multidrug resistance in OSCC by modulating the ABC transporters (106). For decades, small molecule transport inhibitors based on strategies targeting ABC transporters have been developed to avoid drug efflux. Unfortunately, these inhibitors have been proven to have only a limited effect in clinical trials (107, 108). The reason for the failure might be that the inhibitors was targeted and compensatory up-regulation of other ABC transporters could still lead to the efflux of chemotherapeutic drugs. Therefore, a comprehensive study of the ABC transporter family is necessary to understand the function of other proteins in this family. In the meantime, agents that directly target the upstream regulators should be developed to avoid the compensatory mediation between downstream ABC transporters.

#### 2.5.2 Extracellular Vesicles

Extracellular vesicles (Evs) can be subdivided into exosomes (30–100 nm), microvesicles (MVs) (100–1,000 nm), and the oncosomes (1–10 μm), but the way to divide these three by size is not absolute, a more reasonable method is to distinguish them by their origin and function. Extracellular vesicles can deliver cellular cargoes such as proteins, nucleic acids, lipids and other biomolecules or pharmacological compounds to recipient cells, thus affecting the biological functions of recipient cells (109, 110). Currently, exosomes and microvesicles are more studied in OSCC chemoresistance (111, 112). It has been investigated that miR-21 is involved in multidrug resistance of various cancers (113, 114). Liu, et al. found that culturing cisplatin-sensitive OSCC cells with conditioned media obtained from cisplatin-resistant OSCC cells resulted in decreased sensitivity of parental cells to cisplatin and reduced expression of γH2AX, a protein marker characterizing DNA damage. However, application of conditioned media obtained from resistant cells treated with the exosome inhibitor GW4869 reversed this phenomenon. Further mechanistic studies showed that exosomes derived from resistant cells delivered miR-21 to parental cells. MiR-21 targeted PTEN and PDCD4 in cispaltin-sensitive cells, with a resultant inhibition of cisplatin-induced apoptosis (115). Not coincidentally, Chen et al. reported that miR-21 was the most abundantly expressed miRNA in CSC_EVs derived from OSCC cells (43). The application of Ovatodiolide (OV), a newly discovered bioactive component with anti-cancer activity, reduced the expression of oncogenic markers like PI3K, STAT3 and miR-21, and effectively inhibited CSC_EVs mediating cisplatin resistance and tumorigenesis (116). Thus, extracellular vesicles and the cellular cargoes they carry, such as miR-21, can be used as candidate targets for counteracting drug resistance. Moreover, researchers have developed innovative anti-cancer therapies that utilize the biological functions of extracellular vesicles, such as exosomes drug delivery systems (117).

### 2.6 Other Possible Mechanisms of Cisplatin Resistance in OSCC

Epithelial-mesenchymal transition (EMT). Many studies have found that chemo-resistant tumor cells often exhibit a transition from epithelial to mesenchymal, with the disrupted intercellular adhesion, resulting in a greater migratory and invasive capacity of tumor cells and correlating with worse prognosis of patients (105, 118–120). Therefore, blocking the EMT process may improve the antitumor efficacy of chemotherapeutic agents (121, 122). Chen, et al. reported that in OSCC, high expression of FOXD1 induced EMT and resistance to cisplatin *via* the CYTOR/miR-1252-5p/miR-3148/LPP signaling axis, whereas blocking FOXD1 expression could interfere with CYTOR-dependent signaling events, ultimately reducing the EMT phenotype and restoring sensitivity of drug-resistant cell lines to cisplatin (36).

Cancer stem cells (CSCs). According to the cancer stem cell hypothesis, the presence of cancer stem-like cells contributes to tumor recurrence and drug resistance, and there is a correlation between stemness-related genes and poor prognosis (123, 124). CD10 is a newly identified surface marker of cancer-associated fibroblasts, which maintains cancer stemness and facilitates cancer therapy resistance. It was experimentally demonstrated that CD10-positive OSCC cells exhibited enhanced self-renewal ability, tumorigenicity, and poor response to cisplatin. And further studies revealed that CD10 might act through the Hedgehog pathway to enhance cell stemness and cisplatin resistance (125). Naik et al. reported that, in OSCC, cisplatin could induce FaDu cells to exhibit a remarkable stemness feature, and the positive rate of the CSC marker CD44 in cisplatin-resistant FaDu cells reached a peak of 90%. Mechanistically, enhanced autophagy and mitochondrial phagocytosis promoted the accumulation of β-catenin in OSCC cells to activate CSC properties, and thus leading to cisplatin resistance (62).

p53. As a well-known tumor suppressor gene, p53 serves as a hub for cellular stress response by regulating the transcription of multiple downstream targets. p53 mutations are common in human cancers, and mutant p53 not only leads to tumor aggressiveness but also to therapeutic resistance (126, 127). Temam et al. reported that p53 mutations were closely associated with reduced efficiency of platinum- and fluorouracil-based induction chemotherapy in advanced HNSCC (128). In another study, HNSCC cells with cytoplasmic mutant p53 were generally more resistant to cisplatin than cells with nuclear mutant p53. As a result, cytoplasmic p53 mutant proteins promoted upregulation of intracellular ABC transporter proteins (ABCC2 and ABCG2) and increased metabolic activity (129).

Furthermore, several diverse studies about cisplatin resistance in OSCC also merit mention, but the underlying mechanisms do not fall into the aforementioned categories (**Table 2**). For example, a specific region on the endoplasmic reticulum (ER) membrane protein p22phox could bind to cisplatin directly, thus blocking cisplatin entering into the nucleus and inducing DNA damage in OSCC cells (130, 142, 143). Downregulated cylindromatosis (CYLD) contributed to cisplatin resistance of OSCC cells *via* excessive activation of NF-κB (131). Although these findings are mostly inferred from basic experiments, they provide motivation and corroboration for researchers to improve clinical treatment outcomes for OSCC.

**Table 2:** Other factors and cisplatin resistance in OSCC.

Other factors	Expression	Major effects	Reference
p22phox	Up	Blocked cisplatin entering into the nucleus and inducing DNA damage	(130)
Cylindromatosis (CYLD)	Down	Knockdown of CYLD attenuated the cytotoxicity of cisplatin through hyperactivation of NF-κB	(131)
RRBP1	Up	RRBP1 induced cisplatin resistance *via* activating Yes-associated protein1 (YAP1)	(132)
Cancer-derived IgG (CLgG)	Up	Inhibiting the expression of CIgG enhanced cisplatin-induced apoptosis *via* PTP-BAS/Src/PDK1/AKT signaling pathway	(133)
Naa10p	Down	Overexpression of Naa10p enhanced the cisplatin sensitivity in OSCC	(134)
tongue cancer chemotherapy resistance-associated protein1 (TCRP1)	Up	TCRP1 induced the activation of the PI3K/Akt/NF-κB signaling pathway and cisplatin resistance	(135–137)
Yes-associated protein (YAP)	Up	Translocation of YAP from the cytoplasm to the nucleus drived the CDDP resistance in OSCC	(138)
beta-catenin	Up	Overexpressed beta-catenin promoted cisplatin resistance in OSCC	(139)
FAT atypical cadherin 1 (FAT1)		Overexpressed FAT1 inhibited apoptosis vis reducing the oxidative stress and induced cisplatin resistance	(138)
TEAD4	Up	Overexpression of TEAD4 promoted the transcription of S100A13 gene and resulted in chemoresistance	(140)
Nanog	Up	Nanog regulated the expression of Slug, E-cadherin, Oct-4, and c-Myc genes and caused cisplatin resistance	(141)

## 3 Strategies to Reverse Cisplatin Resistance in OSCC and Novel Therapeutic Opportunities

### 3.1 Enhancing Drug Influx: Drug Delivery Systems

Appropriate drug concentration is an important prerequisite to ensure anticancer efficacy. Recently, researchers have attempted to use nanoparticles (NPs) as carriers for chemotherapeutic drug delivery. Owing to ultra-small size and potent adsorption properties, on the one hand, NPs can directly target tumor sites, which results in the localized accumulation of drugs and prolonged drug release time; on the other hand, the targeting of NPs reduces the toxicity of loaded drugs towards normal tissues (144, 145).

Novel N-vinylpyrrolidone (NVP)/acrylic acid (AA) nanoparticles have been revealed to bind and deliver cisplatin into cells *via* coordination bonds. Cisplatin bound to NPs showed higher uptake rate in OSCC cells compared to free cisplatin. This drug delivery system was non-toxic to normal oral epithelial cells, but induced a higher percentage of early apoptosis in OSCC cells and reduced the localized inflammatory response elicited by apoptosis (146). Wang et al. synthesized CDDP loaded and ligand-modified PLGA-PEG/NR7 nanoparticles, which were capable of targeting OSCC cells highly expressing epidermal growth factor receptor (EGFR). Due to the hydrophilic PEG shell and hydrophobic PLGA core of the polymeric NPs, the CDDP loaded therein were allowed for a slow and stable release. In addition, the modified NPs had a higher cellular uptake rate compared to the non-targeting nanoparticles and significantly enhanced the anticancer effect by actively delivering more cisplatin to the tumor site (147).

Overall, nanomedicine delivery systems are promising in enhancing the efficacy of cisplatin and reversing cisplatin resistance in OSCC. Unfortunately, cisplatin-loaded nanoparticles have not yet gained clinical approval.

### 3.2 Combination Therapeutic Regimens

#### 3.2.1 Combination With Cytotoxic Chemotherapeutic Agents

Novel combination regimens involving cisplatin and other cytotoxic agents have been investigated to improve therapeutic outcome of advanced OSCC. Arsenic trioxide (ATO) is one of the few FDA-approved commercially available inorganic non-radioactive anticancer drugs (148), and studies have demonstrated that arsenic trioxide has broad anticancer activity against multiple tumors (149–151). More importantly, the combination of ATO and cisplatin exhibited remarkable synergistic effects (152–154). In OSCC, Kotowski et al. reported that the combination of ATO and CDDP exhibited stronger cytotoxic effects compared to monotherapy (155). Nakaoka et al. found that ATO/CDDP increased the proportion of cells that underwent apoptosis and cell cycle arrest. The potential mechanism could be that ATO/CDDP led to a decrease in mitochondrial membrane potential by stimulating the production of ROS in tumor cells, which subsequently activated the caspase-3/7 signaling pathway, and resulted in cell apoptosis. The experimental results of downregulated cytochrome c, anti-apoptotic Bcl-2 and XIAP also provided a support for this hypothesis (156). However, these results were derived from *in vitro* experiments, and no publications or clinical trials have reported in the subsequent nearly decade that ATO might benefit as a chemosensitizer for cisplatin-based chemotherapy in OSCC, and its efficacy still requires further validation.

With the increasing availability of new cytotoxic agents, investigators have tried a wide range of cisplatin-based combination chemotherapy regimens in clinical trials of OSCC, such as cisplatin plus 5-fluorouracil (PF) combination, cisplatin plus paclitaxel (PP) combination (157), and docetaxel, cisplatin plus 5-FU (TPF) combination (158). However, PP and PF regimens have been proven to show no difference in survival (158). Although TPF regimen has shown superiority over PF regimen and to be favorable in reducing tumor-induced dysphagia and distant metastasis (159), the superiority has been questioned in recent years and there is no definitive evidence that it is superior to concomitant treatment in terms of survival (160–162). In addition, TPF regimens carry a high risk of toxicity, which can be fatal for many patients with co-morbidities, with a treatment-related mortality rate of up to 6% (163). Therefore, the exact role of doublet or triplet chemotherapy regimens requires further exploration, and certain criteria should be established to identify the patient subgroup that would benefit from them, thereby improving treatment outcomes and reducing treatment risks.

#### 3.2.2 Combination With Molecular Targeted Agents

In studies of OSCC tumor heterogeneity, a number of genes and molecules associated with poor prognosis have been identified to be commonly altered, and researchers view them as potential targets for OSCC treatment.

EGFR is a member of the human epidermal growth factor receptor (HER) family. Currently, there are two main types of medications that target EGFR in clinical practice, which are monoclonal antibodies represented by cetuximab and tyrosine kinase inhibitors (TKIs) (164). In a multi‐centric phase III clinical trial, a regimen of cetuximab combined with platinum–fluorouracil improved treatment response rate, PFS and OS in patients with recurrent and/or metastatic squamous cell carcinoma of the head and neck (R/M-SCCHN) compared with platinum–fluorouracil chemotherapy alone (165). However, the benefits of the combined regime was not absolute, in another phase III clinical trial, the addition of cetuximab failed to improve PFS or OS in patients with stage III-IV OSCC receiving cisplatin and radiation therapy (166). When it comes to EGFR-TKIs, regrettably, there was insufficient evidence showing the addition of EGFR-TKIs to standard therapies could provide overall therapeutic benefits. Additionally, cetuximab resistance has been reported, and the use of EGFR-mAb and EGFR-TKIs may lead to increased skin toxicity (167, 168).

Apart from EGFR, targeting other molecules can also benefit the anticancer treatment. Stathmin is a downstream molecule in the PI3K/AKT/mTOR signaling pathway, and OSCC patients with high stathmin expression responded poorly to TPF chemotherapy regimens. The combination of PI3K inhibitor, BKM-120, with TPF decreased the expression and phosphorylation of stathmin, and induced cell cycle arrest, thereby increasing the proportion of apoptotic tumor cells and inhibiting the growth of xenografts (169). The combination of Bruton’s tyrosine kinase (BTK) inhibitors, ibrutinib, and cisplatin were capable of suppressing stemness characteristics and promoting apoptosis in OSCC cells (170).

Since the above findings are mostly obtained from prospective studies, the simultaneous or sequential combination of targeted agents and conventional chemotherapeutic agents remains controversial, and the combination strategies need further clinical study.

#### 3.2.3 Combination With Immunotherapeutic Drugs

Recent studies have shown that early immunotherapy is of great benefit to the prognosis of OSCC patients. New generation of immunotherapy applies immune checkpoint inhibitors to alter the immunosuppressive tumor microenvironment and reactivate the host immune response to tumor cells, thereby inhibiting tumor cell proliferation and even triggering apoptosis (171). In 2016, two immune checkpoint inhibitors, pembrolizumab and nivolumab, were approved by FDA for advanced R/M-SCCHN patients who had disease progression during or following treatment with cisplatin (172–174). Following that, multiple immune checkpoint inhibitors (ICIs) have been applied to the treatment of OSCC, among which PD-1/PD-L1 targeted are the most numerous. In a systematic review of eight clinical trials applying ICIs, the authors reported that the standard therapy had poor efficacy in PD-L1-positive R/M SCCHN patients, while the immunotherapy had a better performance. The overall survival (OS) of R/M SCCHN patients using PD-1 checkpoint inhibitors ranged from 7.5 to 14.9 months, which was significantly longer compared to those receiving standard therapy, and the life quality of patients was significantly improved due to immunotherapy producing fewer systemic toxicities (175). Immune checkpoint inhibitors and cisplatin exert anticancer activity through different mechanisms, and immunochemotherapy combining the two may be beneficial in the treatment of advanced OSCC. However, Tringale et al. reported that the overall efficiency of current immune checkpoint inhibitors for R/M SCCHN is not favorable due to the high cost of treatment, and further research is needed to minimize treatment costs and improve treatment outcomes (176).

#### 3.2.4 Combination With Potential Anti-Cancer Drugs: Natural Products

Since a long time, natural products have served as one of the most important sources of anti-cancer drugs. Some anticancer drugs that possess definite anticancer activity and have been widely used, such as hydroxycamptothecin and paclitaxel (PTX), are natural products, secondary metabolites and/or their structural analogues. Recently, combination therapies of natural products and traditional anticancer drugs have shown potential on improving therapeutic effect.

Sulforaphane (SF) and curcumin (CRM) are both natural compounds derived from plants and commonly employed as antioxidants in anticancer therapy (177). The combination of SF and CDDP inhibited the growth of SCCHN cells and made the cytotoxicity of CDDP 2 times magnified (178). While the combination therapy with liposomal curcumin and cisplatin also had an enhanced growth inhibitory effect on HNSCC compared to monotherapy (179). In addition, several natural products, ursolic acid (180), plumbagin (181) and epigallocatechin gallate (182) are also worth mentioning, which exhibit chemosensitizing function in cisplatin-resistant OSCC through similar mechanisms. Although the potential of natural products in anticancer therapy has been demonstrated in many preclinical trials, there are still many hurdles to overcome before formal clinical application, such as the low water solubility, poor stability, and low oral availability. Therefore, the physicochemical characterization of natural products needs to be further improved in the future to achieve effective clinical application.

## 4 Conclusion and Perspectives

Resistance to cisplatin remains a major challenge that hinders the success of OSCC treatment. According to current knowledge, multiple factors such as DNA damage and repair, transport process, and programmed cell death are involved in initial or acquired resistance to platinum-based drugs in OSCC. Recently, new factors such as epigenetic biological processes and tumor microenvironment have also received increasing attention in the study of the mechanisms of chemoresistance in OSCC. Increased understanding of the underlying molecular mechanisms of cisplatin resistance is essential to predict, prevent, and reverse chemoresistance. Drug resistance is essentially a form of tumor heterogeneity, and the molecules that have been reported to be associated with OSCC cisplatin resistance may not be representative of the entire resistant subset due to small sample size or lack of clinical validation. In the future, a large-scale screening may be needed to identify biomarkers associated with cisplatin resistance in OSCC and validate them prospectively before treatment, thus identifying cases that may benefit from cisplatin-based treatment and providing more rational personalized treatment regimens for resistant patients, which could improve treatment outcomes and increase survival in refractory OSCC patients.

## Author Contributions

SML and LG gathered the related literature. YLC drafted the first manuscript and created the graphs. KQZ and WHR designed this review and contributed to critical revision of the manuscript. All authors contributed to the article and approved the submitted version.

## Funding

This work was supported by the Postdoctoral Science Foundation of China (2017M622145), Natural Science Foundation of Shandong Province (ZR2018BH021), Science and Technology Project of Qingdao West Coast New Area (2019–60).

## Conflict of Interest

The authors declare that the research was conducted in the absence of any commercial or financial relationships that could be construed as a potential conflict of interest.

## Publisher’s Note

All claims expressed in this article are solely those of the authors and do not necessarily represent those of their affiliated organizations, or those of the publisher, the editors and the reviewers. Any product that may be evaluated in this article, or claim that may be made by its manufacturer, is not guaranteed or endorsed by the publisher.
